# ﻿A new species and a replacement name in *Cynanchum* (Apocynaceae, Asclepiadeae) from China

**DOI:** 10.3897/phytokeys.241.111499

**Published:** 2024-04-08

**Authors:** Miao Liao, Si-Jin Zeng, Lin-Ya Zeng, Hai-Jun Yin, Mao-Lin Yan, Cai-Fei Zhang, Guang-Da Tang

**Affiliations:** 1 College of Forestry and Landscape Architecture, South China Limestone Plants Research Center, South China Agricultural University, Guangzhou, China South China Agricultural University Guangzhou China; 2 CAS Key Laboratory of Plant Germplasm Enhancement and Specialty Agriculture, Wuhan Botanical Garden, Chinese Academy of Sciences, Wuhan, China Wuhan Botanical Garden, Chinese Academy of Sciences Wuhan China; 3 Sino-Africa Joint Research Center, Chinese Academy of Sciences, Wuhan, China Sino-Africa Joint Research Center, Chinese Academy of Sciences Wuhan China; 4 University of Chinese Academy of Sciences, Beijing, China University of Chinese Academy of Sciences Beijing China; 5 State Key Laboratory of Plant Diversity and Specialty Crops/Key Laboratory of Plant Resources Conservation and Sustainable Utilization, South China Botanical Garden, Chinese Academy of Sciences, Guangzhou, China South China Botanical Garden, Chinese Academy of Sciences Guangzhou China; 6 Tongbiguan Provincial Nature Reserve, Ruli, Yunnan, China Tongbiguan Provincial Nature Reserve Ruli China; 7 Henry Fok College of Biology and Agriculture, Shaoguan University, Shaoguan, China Shaoguan University Shaoguan China

**Keywords:** *Cynanchumhooperianum*, *Cynanchumlonghushanense*, *Cynanchumpingtaoi*, morphology, phylogeny, *Raphistemma*, taxonomy

## Abstract

*Cynanchumpingtaoi* S.Jin Zeng, G.D.Tang & Miao Liao, **sp. nov.** (Apocynaceae) from Yunnan Province, China, is described and illustrated based on morphological and molecular evidence. Its deeply cordate to reniform leaves and campanulate, large flowers show that it is a member of former *Raphistemma* Wall., which has been included in *Cynanchum* L.. It is different from all former *Raphistemma* species by the broadly ovate corolla lobes, purple-red corolla and connivent corona tip slightly exceeding the corolla throat. Meanwhile, *Cynanchumlonghushanense* G.D.Tang & Miao Liao, **nom. nov.** is proposed as replacement name for *Raphistemmabrevipedunculatum* Y.Wan, which was considered a synonym of *Cynanchumhooperianum* (Blume) Liede & Khanum but is here reinstated as a distinct species because of significant morphological differences.

## ﻿Introduction

*Cynanchum* L. is one of the largest genera of Asclepiadeae (Apocynaceae) and is mainly distributed in Africa and Asia, but it occurs as well in the New World (Khanum et al. 2016; Endress et al. 2018). It is considered a “dustbin genus” due to its complex morphological characteristics, but species generally possess a gynostegial, at least basally fused corona (Liede and Kunze 1993). The extent and classification of *Cynanchum* are controversial. Markgraf (1972) and Ali and Khatoon (1982) supported *Cynanchum* and *Vincetoxicum* Wolf as separate. However, other studies did not support this circumscription (Tsiang and Li 1977; Li et al. 1995). Woodson (1941) and Liede (1997) suggested *Cynanchum* should include more than twenty allied genera but not *Vincetoxicum*. Recent studies, based on molecular and morphological evidence, changed the *Cynanchum* circumscription, including more than ten genera (e.g., *Raphistemma* Wall.) (Khanum et al. 2016) and segregating *Vincetoxicum* (Qiu et al. 1989; Liede 1996, 2001; Rapini et al. 2007). A wide concept of the genus with about 250 spp. has been accepted (Khanum et al. 2016; Endress et al. 2018), and the newly circumscribed *Cynanchum* generally comprises species which possess a corolla 3–10 (–40) mm long, a highly variable gynostegial corona, usually with a ring of fused staminal and interstaminal parts, the staminal parts occasionally with an adaxial appendage, occasionally with additional free staminal lobes connate to ring (Endress et al. 2018).

*Cynanchum* is a large and complex genus, and the Chinese representatives of *Cynanchum* have not been completely revised since the “Flora of China” (Li et al. 1995). The number of species of Chinese *Cynanchum* recorded in databases and literature references differs considerably, e.g., “Flora of China” reports 57 species of *Cynanchum* in China (Li et al. 1995); Plants of the World Online (POWO, https://powo.science.kew.org/) cites 45 species (including *C.heydei* Hook.f. distributed in Xizang, China and *C.lanhsuense* T.Yamaz. which occurs only in Taiwan, China); Catalogue of Life China 2023 Annual Checklist (http://www.sp2000.org.cn/CoLChina) cites 44 species; and Xu et al. (2021) cite 45 species. Of the 57 species of *Cynanchum* reported by “Flora of China”, 26 species now belong to *Vincetoxicum* (Liu 2023; POWO 2024) (Suppl. material 1) and *Cynanchum* includes nine species originally housed in allied genera, notably *Adelostemmagracillimum* (Wall. ex Wight) Hook.f., *Graphistemmapictum* (Champ. ex Benth.) Maxim., *Holostemmaada-kodien* Schult., *Metaplexishemsleyana* Oliv., *M.japonica* (Thunb.) Makino, *Raphistemmahooperianum* (Blume) Decne., *R.pulchellum* (Roxb.) Wall., *Sichuaniaalterniloba* M.G.Gilbert & P.T.Li. (Khanum et al. 2016), and *Sarcostemmaacidum* (Roxb.) Voigt (Liede and Kunze 2002; Liede and Täuber 2002). Besides, three species were missed by the authors of “Flora of China”, which are *C.defilippii* Delponte (Delponte 1871), *C.kaschgaricum* Y.X.Liou (Liou et al. 1992) and *C.lanhsuense* T.Yamaz. (Yamazaki 1968). Also, recent studies described three new species of *Cynanchum* from China (Grubov 2000; Shen et al. 2019; Xu et al. 2021). Currently, 46 species of *Cynanchum* occur in China.

During a field survey in 2020, we collected an unknown species in Ruili, Yunnan. This species has the typical characters of former *Raphistemma* species with large and reniform leaves, and large and campanulate corollas. Four names were recorded in *Raphistemma*: *R.ciliata* Hook.f. was treated as a synonym of *Pergulariadaemia* (Forssk.) Chiov. (Goyder 2006); *R.pulchellum* was revised to *C.pulchellum* (Roxb.) Liede & Khanum (Khanum et al. 2016); *R.brevipedunculatum* Y.Wan was considered a synonym of *R.hooperianum*, which has been revised to *C.hooperianum* (Blume) Liede & Khanum (Li et al. 1995; Khanum et al. 2016). After careful examination of the specimens and literature of former *Raphistemma*, we are unable to match the newly collected material with any recorded species. Therefore, we here describe the newly collected material as a new species of *Cynanchum*. Meanwhile, we found significant morphological differences between *R.brevipedunculatum* and *R.hooperianum*. We therefore propose to reinstate *Raphistemmabrevipedunculatum* at species level in the genus *Cynanchum*: since the name is already in use within the genus, a replacement name is necessary and proposed here.

## ﻿Material and methods

### ﻿Morphological observation

For the new species, field observations were done and a collection made in Ruili City, Yunnan Province (specimen voucher: *Si-Jin Zeng & Lin-Ya Zeng SJ4825* (IBSC)). We collected a living sample of *R.brevipedunculatum* from the type locality (Longhushan Nature Reserve, Longan County, Guangxi Zhuang Autonomous Region (specimen voucher: *Miao Liao LM78* (IBSC)). Literature referring to *Cynanchumpulchellum*, *C.hooperianum*, and *R.brevipedunculatum* was consulted, including protologues (Blume 1826; Wan 1983) and other literature (Wallich 1831; Decaisne 1844; Backer and Bakhuizen van den Brink 1965; Li et al. 1995; Khanum et al. 2016). Specimens of *C.hooperianum*, *C.pulchellum*, and *R.brevipedunculatum* at CANT, GXMI, HIB, IBSC (herbaria acronyms follow Thiers 2022) and specimen images from the Chinese Virtual Herbarium (CVH, http://www.cvh.ac.cn), GXSP, National Specimen Information Infrastructure (NSII, http://www.nsii.org.cn), the Global Biodiversity Information Facility (GBIF, https://www.gbif.org/), the JSTOR Global Plants database (https://plants.jstor.org/), P (http://science.mnhn.fr/institution/mnhn/collection/p/item/search/form), and TI (http://umdb.um.u-tokyo.ac.jp/DShokubu) were checked. Morphological data of *R.brevipedunculatum* is based on living plants, specimens collected in the field and protologues (Wan 1983). Characteristics of *C.hooperianum* are taken from Decaisne (1844) and Backer and Bakhuizen van den Brink (1965) and those of *C.pulchellum* from Wallich (1831) and Backer and Bakhuizen van den Brink (1965).

### ﻿Phylogenetic analysis

#### ﻿DNA extraction, sequencing, assembly, and annotation

We obtained total genomic DNA of the new species and *R.brevipedunculatum* from fresh leaf material dried in silica gel with the plant genomic DNA kit (DP305, Tiangen, Beijing, China). The samples were sent to Novogene (Tianjin, China) for library preparation (350 bp) for genome skimming sequencing. We used an Illumina HiSeq 2000 to conduct a paired-end sequencing (150 bp), generating 10 Gb raw data for each sample. After quality control of the raw data using fastp 0.19.7 (Chen et al. 2018: parameter settings fastp -g -q 5 -u 50 -n 15 -l 150), we extracted 3 Gb paired reads for plastid and nuclear ribosome assembly using GetOrganelle v.1.7 with the parameters “-t 30 -R 15 -k 21,45,105,115,127 -F embplant_pt” and “-t 30 -R 7 -k 35,85,115 -F embplant_nr” respectively (Jin et al. 2020).

A plastid genome of *Apocynumvenetum* L., a continuous sequence (18S-ITS1-5.8S-ITS2-26S) of the ribosome genome of *Asclepiasalbicans* S.Watson, and the nrETS of *Calciphilagalgalensis* (Liede) Liede & Meve and *Cynanchumadalinae* (K.Schum.) K.Schum. (GenBank accession numbers: MT313688, JN665082, LN896997, and LN897003) were used as respective references. We employed Geneious Prime 2019 (https://www.geneious.com) to annotate and extract three plastid DNA markers (one spacer of *trnL-F* and two introns of *rps16* and *trnL*) and two nuclear DNA regions (nrITS: internal transcribed spacer; nrETS: external transcribed spacer). New sequences of the five loci were uploaded to GenBank with accession numbers OP810602–OP810613 and OP853101–OP853103 (https://www.ncbi.nlm.nih.gov/).

#### ﻿Taxa sampling, alignment, and phylogenetic analysis

Sequences of the new species and *R.brevipedunculatum* were added to a reduced matrix of Khanum et al. (2016) with combined data of five loci (nrETS, nrITS, *rps16*, *trnL*, and *trnL-F*) for phylogenetic analysis (Suppl. material 2). We used 57 samples for molecular analysis, including 50 samples (49 species) of *Cynanchum*, covering all nine clades in Khanum et al. (2016). Of the former *Raphistemma* species, we included *C.pulchellum* and *C.hooperianum* (in fact a specimen belonging to *R.brevipedunculatum*). Seven samples were taken as outgroups: *Pentatropismadagascariensis* Decne., *P.nivalis* (J.F.Gmel.) D.V.Field & J.R.I.Wood, and *P.* sp. which belong to subtribe Tylophorinae, and *Calciphilagalgalensis* (including two samples), *Calciphilagillettii* Liede & Meve and *Calotropisprocera* (Aiton) W.T.Aiton which belong to subtribe Asclepiadinae.

We aligned sequences of the three plastid markers and two nuclear regions separately using the MUSCLE optional in the software MEGA v.7.0.26 (Kumar et al. 2016) and concatenated the data using the Concatenate Sequences or Alignments optional in Geneious Prime 2019 (https://www.geneious.com). The incomplete sequences were filled with missing data.

We used RAxML-HPC2 8.2.12 on XSEDE (Stamatakis 2014) through the CIPRES portal (Miller et al. 2010) to perform Maximum likelihood (ML) analysis by using the GTRCAT model and 1 000 bootstraps. Finally, we viewed and edited the tree in Figtree 1.4.2 (Rambaut 2012).

## ﻿Results

### ﻿Morphological comparison

The new species resembles *Cynanchumhooperianum*, *C.pulchellum*, and *Raphistemmabrevipedunculatum*, but differs from them by the broadly ovate corolla lobes, purple-red corolla, and connivent corona apex slightly exceeding the corolla throat (Table 1).

**Table 1.** Morphological comparison of *Cynanchumpingtaoi* and closely related species.

Characters	*Cynanchumpingtaoi*	*Cynanchumhooperianum*	*Cynanchumpulchellum*	*Cynanchumlonghushanense* (*Raphistemmabrevipedunculatum*)
Calyx length	ca. 6 mm	3–4 mm	3–4 mm	5–6 mm
Corolla-tube length	12–14 mm	8–9 mm	12–18 mm	12–16 mm
Corolla lobe shape	Broadly ovate (Fig. 4h)	Ovate	Ovate-oblong	Oblong (Fig. 2i)
Corolla color	Outer surface greenish-white, inner surface purple-red (Fig. 4h)	Outer surface light green, inner surface white, with purple spots near the top of corolla lobe	Outer surface slightly greenish, inner surface white	Outer surface greenish-white, inner surface white (Fig. 2i)
Corona-scales apex	Connivent, corona apex slightly exceeding corolla throat (Fig. 3e)	Separate, reaching the middle of the corolla-segments or further	Barely connivent, slightly exceeding corolla throat	Connivent, not exceeding corolla throat (Fig. 2h)
Follicles	Fusiform, with three ribs	Not observed	Oblong, without ribs	Fusiform, with three ribs

*Raphistemmabrevipedunculatum* is significantly different from *Cynanchumhooperianum* by the longer calyx lobes (5–6 mm vs. 3–4 mm), the longer corolla-tubes (12–16 mm vs. 8–9 mm), the oblong corolla lobes (vs. ovate), and the corona lobes with connivent corona-scales apex, not exceeding the throat of the corolla (vs. corona-scales apex separate, reaching the middle of the corolla-segments or further). *Raphistemmabrevipedunculatum* flowers in June–July (Wan 1983), but *Cynanchumhooperianum* flowers almost all year (Blume 1826).

### ﻿Molecular phylogenetics

**Figure 1.**
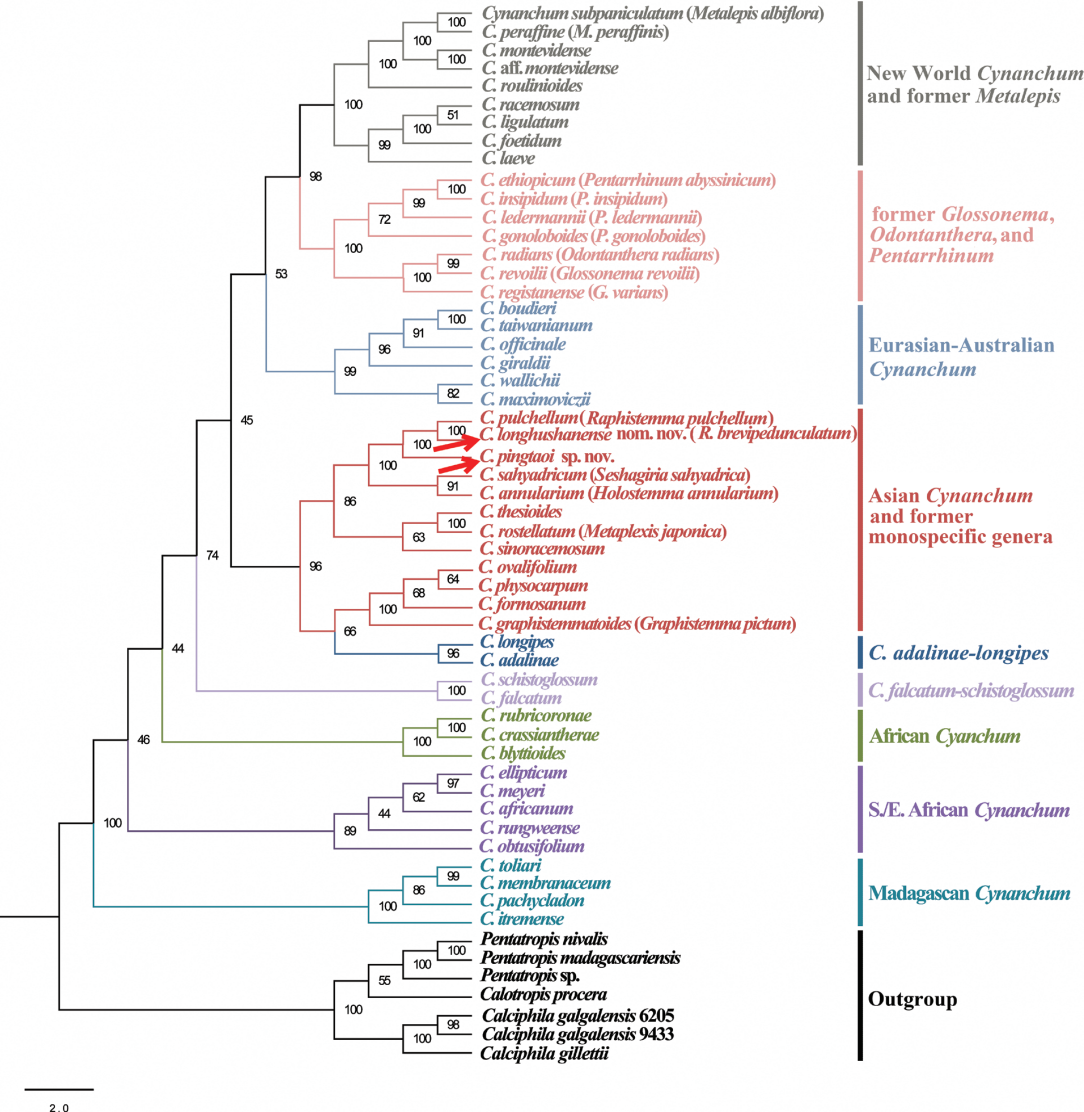
Simplified maximum likelihood tree of *Cynanchum* based on two nuclear regions (nrETS and nrITS) and three plastid markers (*rps16* and *trnL* introns, and *trnL-F* spacer). Bootstrap support values are given for each node. See Suppl. material 3 for maximum likelihood tree. Clade designations follow those of Khanum et al. (2016) and names in parentheses are the corresponding names that were used in Khanum et al. (2016).

The combined phylogenetic analysis shows that the new species forms a monophyletic clade (BS = 100%, Fig. 1, Suppl. material 3) with two former *Raphistemma* species. The two former *Raphistemma* species (*C.pulchellum* and *R.brevipedunculatum*) are sister species (BS = 100%, Fig. 1, Suppl. material 3).

### ﻿Taxonomic treatments

#### ﻿ Cynanchum longhushanense

Taxon classificationPlantaeGentianalesApocynaceae

G.D.Tang & Miao Liao, nom. nov., non Cynanchum brevipedunculatum J.Y.Shen et al. (2019: 217).

6743861D-8025-5C65-BEF2-15D966F7B361

urn:lsid:ipni.org:names:77339615-1

Fig. 2

≡ Raphistemmabrevipedunculatum Y.Wan, in Guihaia 3(3): 197 (1983).

##### Type.

China. Guangxi: Longan County, Longhushan Nature Reserve, open woods, 2 Jul. 1981, *D.H. Tan 81329* (holotype: GXSP[GXSP0000038!]; isotype: CANT[CANT00002128!]).

##### Chinese name.

Guangxi Dahuateng (广西大花藤).

##### Notes.

**Figure 2.**
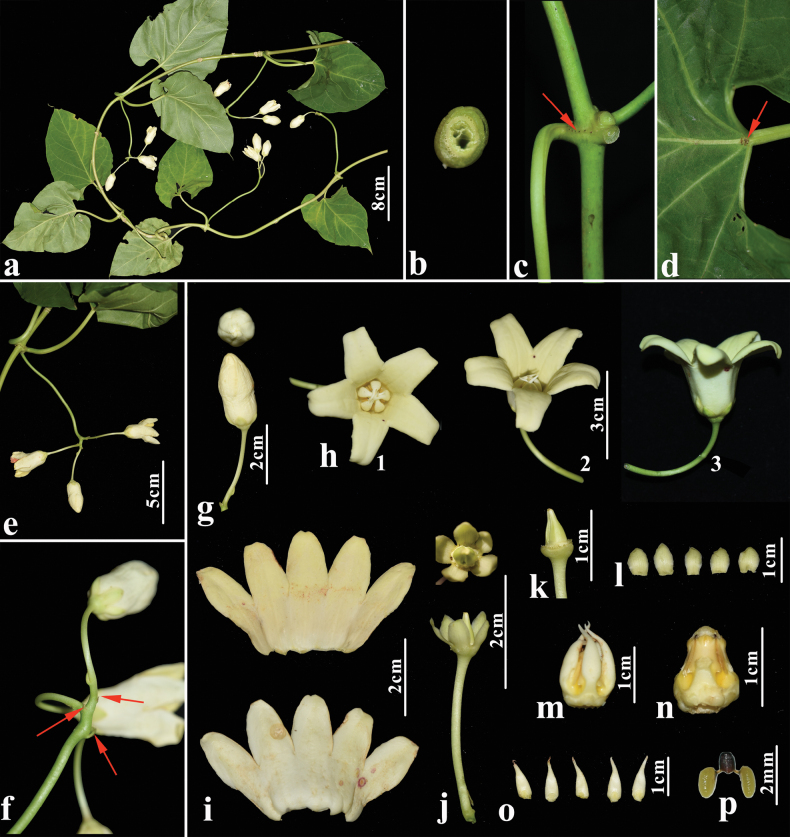
*Cynanchumlonghushanense* G.D.Tang & Miao Liao **a** flowering branch **b** cross-section of hollow stem **c** node with small glands **d** petiole with glands at the top **e** inflorescence **f** bracteoles at the base of the pedicel **g** flower bud (above: top view; below: side view) **h** flower (1) top view, (2) lateral view, showing the connivent corona-scales apex, not exceeding the throat of the corolla, (3) side view, showing corolla lobes overlapping to the right and corolla tube **i** opened corolla, adaxial (above) and abaxial (below) view **j** ovary with calyx (above: top view, showing glands at the base of the calyx, below: side view of ovary, calyx and pedicel) **k** ovary **l** calyx lobes **m** gynostegium with corona lobes **n** gynostegium **o** corona lobes **p** pollinarium. All photos based on *Miao Liao LM78*.

*Raphistemmabrevipedunculatum* was considered a synonym of *R.hooperianum* (Li et al. 1995), which was transferred to *Cynanchum* as *C.hooperianum* (Khanum et al. 2016). Although *C.hooperianum* is not included in our molecular study, morphological differences between specimens of *C.hooperianum* and *R.brevipedunculatum* are significant. We therefore reinstate *R.brevipedunculatum* as a distinct species of *Cynanchum*. We renamed *Raphistemmabrevipedunculatum* as *Cynanchumlonghushanense* because the specific epithet ‘*brevipedunculatum*’ is occupied in *Cynanchum* (*C.brevipedunculatum* J.Y.Shen). *C.longhushanense* differs from *C.hooperianum* by the longer calyx lobes (5–6 mm vs. 3–4 mm), the longer corolla-tubes (12–16 mm vs. 8–9 mm), the oblong corolla lobes (vs. ovate), and the corona lobes with connivent corona-scales apex, not exceeding the throat of the corolla (vs. corona-scales apex separate, reaching the middle of the corolla-segments or further). It should be noticed that in the protologue of *Cynanchumlonghushanense* (≡ *R.brevipedunculatum*) (Wan 1983), the number of glands at the base of the calyx was described as five, but we observed 10–15 glands per calyx in our Guangxi collection (Fig. 2j). More specimens are expected to be collected to determine the morphological variation.

##### Other specimens examined.

China. Guangxi: Longan County, Pingshan Village, 10 Oct. 1977, *Longan Investigation Team 2-040* (GXMI[GXMI031735!]); Longan County, Longhushan Nature Reserve, 14 Nov. 1982, *Y. Wan & Rui-Ju Liu 82430* (paratype: GXSP[GXSP0000039!]); Longan County, Longhushan Nature Reserve, 25 Jun. 2021, *Miao Liao LM78* (IBSC).

#### ﻿ Cynanchum pingtaoi

Taxon classificationPlantaeGentianalesApocynaceae

S.Jin Zeng, G.D.Tang & Miao Liao sp. nov.

45581333-BF45-5562-808C-98F7EB1C761F

urn:lsid:ipni.org:names:77339616-1

Figs 3 , 4

##### Type.

China. Yunnan: Dehong Dai and Jingpo Autonomous Prefecture, Ruili City, Nongdao Town, Tongbiguan Provincial Nature Reserve, 23°57'N, 97°32'E, elev. 839 m, 24 Aug. 2020, *Si-Jin Zeng & Lin-Ya Zeng SJ4825* (holotype, IBSC[IBSC1009908!]; isotypes, IBSC [IBSC1009907!], [IBSC1009909!], [IBSC1009910!]).

##### Diagnosis.

*Cynanchumpingtaoi* resembles *C.longhushanense*, differing by its broadly ovate corolla lobes (vs. oblong), the purple-red inner surface of the corolla (vs. white), and the corona-scales apex connivent, slightly exceeding the corolla throat (vs. corona-scales apex connivent, not exceeding the throat of the corolla).

**Figure 3.**
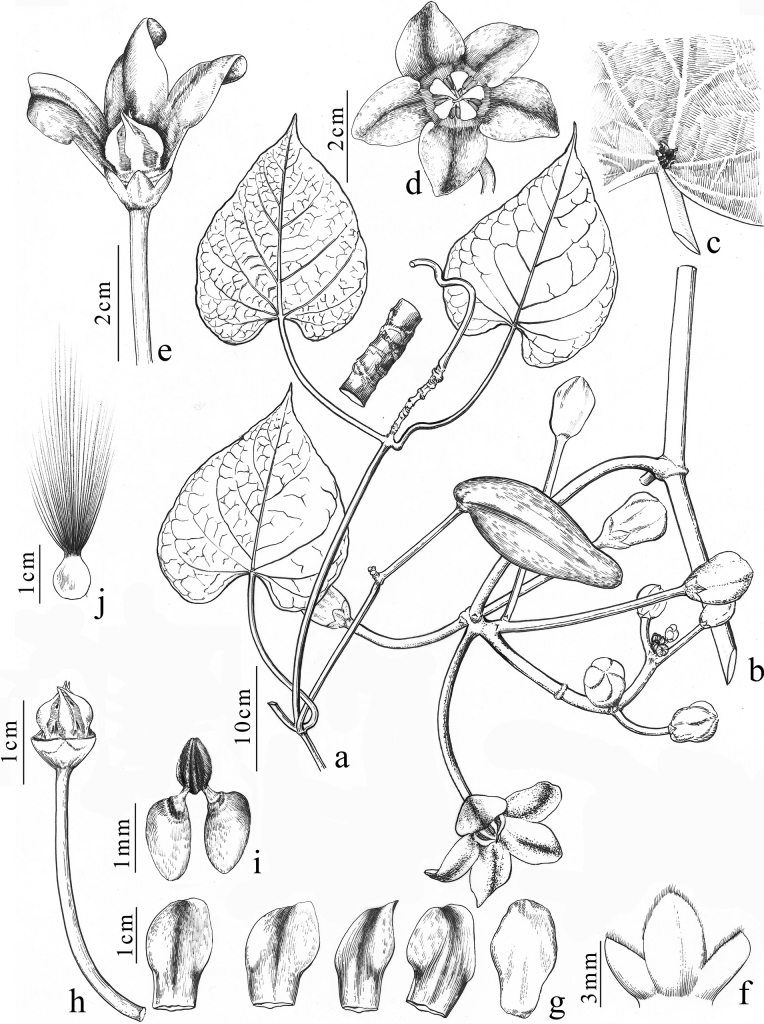
*Cynanchumpingtaoi* S.Jin Zeng, G.D.Tang & Miao Liao **a** follicle and leaves **b** inflorescence **c** glands at the base of leaf **d** top view of flower **e** side view of flower with two corolla lobes removed **f** calyx **g** corolla lobes (each lobe attached to part of the corolla tube), outer surface on the right, inner surface on the left (four drawn) **h** corolla separation, showing gynostegium with corona lobes **i** pollinarium **j** seed. Illustration based on *Si-Jin Zeng & Lin-Ya Zeng SJ4825* (IBSC), and drawn by Ding-Han Cui.

##### Description.

Twining liana. White latex in stems and leaves. **Branchlets** fistulous, smooth, glabrous, slightly woody. **Leaves** opposite; petiole 6–14 cm long, smooth, sparsely white puberulent, later glabrescent, with small yellowish-brown glands at the apex, nodes with small glands; leaf blade deeply cordate to reniform, 7–15 × 4–13 cm, membranous, base cordate, apex acuminate, margin entire, adaxial surface dark green, glabrous, abaxial surface light green, sparsely white puberulent on veins, gradually glabrescent later; basal veins five or seven, palmate, secondary veins three to five pairs, pinnate, tertiary veins reticulate, smooth adaxially, raised abaxially. **Inflorescences** extra-axillary, subumbellate to subracemic, 5–11 flowers; peduncle 10–12 cm long, smooth; pedicel 3.0–5.5 cm long, smooth, sparsely white puberulent near the base, base with bracteoles triangular, ca. 0.1 × 0.1 cm. **Calyx** yellowish green, basally fused, lobes elliptic, ca. 0.6 × 0.5 cm, inside the base with small glands, apices obtuse, margins ciliate. **Corolla** campanulate, glabrous, external surface greenish white, inner surface purple-red, 3.3–3.5 cm in diam; lobes slightly longer than tube, tube 1.2–1.4 cm, lobes broadly ovate, 1.3–1.8 × 1–1.2 cm, apices reflexed, overlapping to the right. **Corona** lobes linear-subulate, white, separate, ca. 1.1 cm long, inserted at base of gynostegium, longer than gynostegium, corona-scales apex connivent, slightly exceeding corolla throat. **Anthers** ca. 0.7 × 0.4 cm, **apices** with inwardly incurved wings. **Stigma** broadly rounded, slightly depressed, white. **Pollinia** 2 per pollinarium, ellipsoid, yellow, pendulous, ca. 0.13 × 0.08 cm, caudicle ca. 0.05 cm long, retinaculum ca. 0.1 cm long. **Follicles** solitary, fusiform, ca. 14.5 cm long, ca. 5 cm diam., glabrous, with a thick fibrous pericarp, triangulate, apex curved outwards; seeds ovoid, 0.8 cm × 0.6 cm, tipped with a white silky coma; coma 3.8–4.2 cm long.

**Figure 4.**
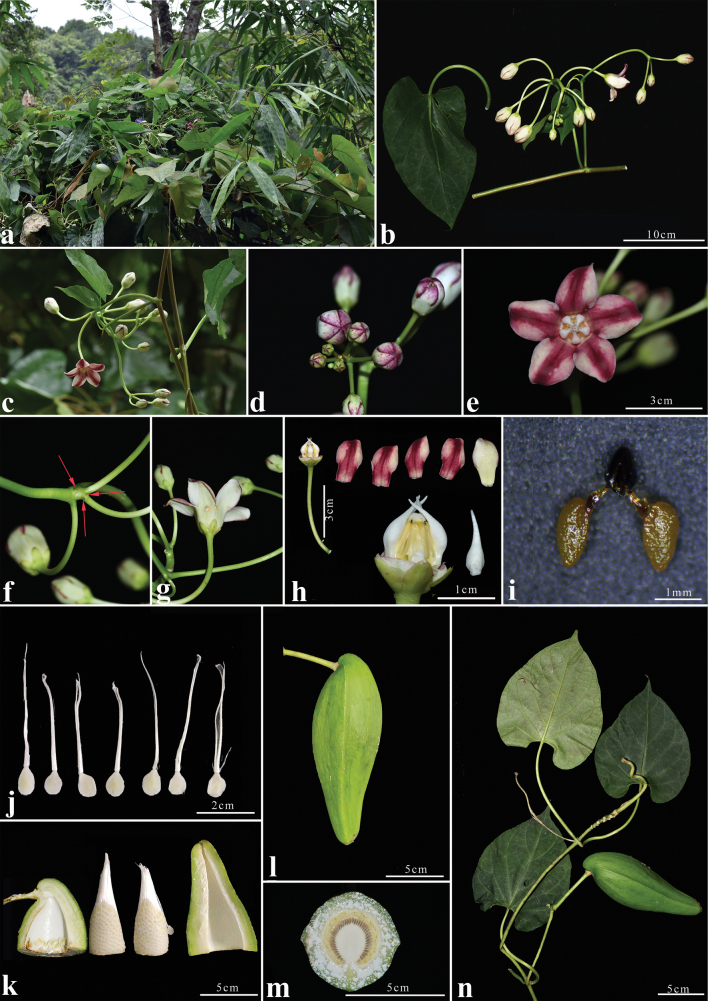
*Cynanchumpingtaoi* S.Jin Zeng, G.D.Tang & Miao Liao **a** habitat **b** inflorescence and leaf **c** inflorescence **d** flower buds **e** top view of flower **f** bracteoles at base of pedicel **g** side view of flower **h** corolla and corona separation, showing gynostegium, corona and corolla lobes (outer surface: upper right hand corner; inner surface: to the left of the outer surface) **i** pollinarium from dry specimen **j** young seeds **k** longitudinally opened half follicle showing seed arrangement **l** follicle, different views to show the three ribs **m** transverse section through follicle **n** follicle and leaves. All photos based on *Si-Jin Zeng & Lin-Ya Zeng SJ4825* (IBSC).

##### Etymology.

The specific epithet *pingtaoi* honors the eminent botanist Ping-Tao Li (李秉滔), who is an expert in the Apocynaceae.

##### Chinese name.

Bingtao Dahuateng (秉滔大花藤).

##### Distribution.

**Figure 5.**
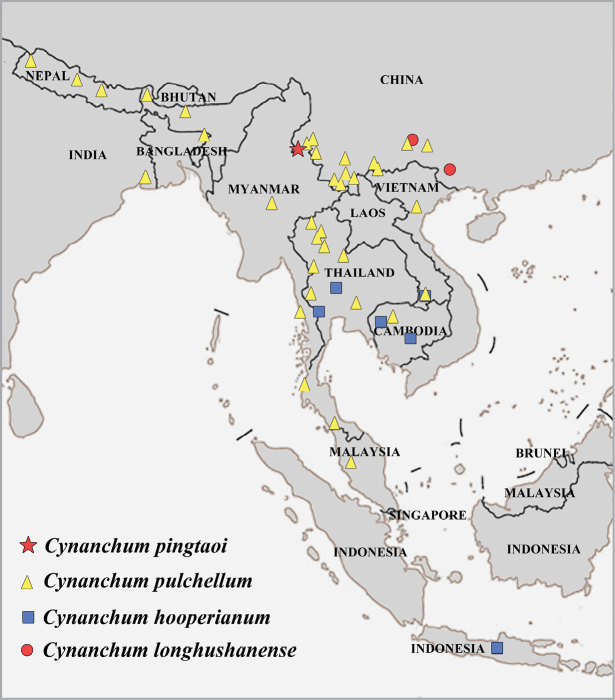
Distribution map of *Cynanchumpingtaoi* and the three *Cynanchum* species formerly were considered to belong to *Raphistemma* based on specimens cited in Suppl. material 4. The base map [No. GS(2016)1666] was downloaded from http://bzdt.ch.mnr.gov.cn/.

Endemic to China. Only one population was found at the border of China-Myanmar in Ruili, Yunnan Province, China. Fig. 5.

##### Habitat and phenology.

This species occurs near open woods at an elevation of about 850 m. Flowering was observed from September to October, fruiting from November to December.

##### Provisional IUCN assessment.

The species is currently known only from the type locality, where only a few individuals were seen. Suitable habitat exists in the proximity of the type locality. Nevertheless, as there is no reliable information on the population size or distribution of this species, we propose to treat it as Data Deficient (IUCN 2019).

##### Notes.

The large and reniform leaves, campanulate corolla, and lanceolate corona indicate that this species is morphologically close to the former *Raphistemma* species, which have been included in *Cynanchum* (Khanum et al. 2016). The molecular phylogenetic analyses also showed that *Cynanchumpingtaoi* is sister to *C.pulchellum* + *C.longhushanense* (Fig. 1; Suppl. material 3). We do not currently possess molecular data of *C.hooperianum*, but the new species can be distinguished from this species by the purple-red inner surface of the corolla (vs. white inner surface of the corolla with purple spots near the top of the corolla lobes), the connivent corona-scales apex, slightly exceeding the corolla throat (vs. corona-scales apex separate, and reaching the middle of the corolla-segments or somewhat further), the longer calyx-segments (6 mm vs. 3–4 mm), and the longer corolla-tubes (12–14 mm vs. 8–9 mm).

## Supplementary Material

XML Treatment for Cynanchum longhushanense

XML Treatment for Cynanchum pingtaoi

## ﻿Additional information

### Author ORCIDs

Miao Liao https://orcid.org/0000-0003-2545-9419

Si-Jin Zeng https://orcid.org/0000-0002-3256-0286

Lin-Ya Zeng https://orcid.org/0000-0002-4788-1867

Hai-Jun Yin https://orcid.org/0009-0004-9804-1958

Mao-Lin Yan https://orcid.org/0009-0003-5188-6796

Cai-Fei Zhang https://orcid.org/0000-0002-2818-5751

Guang-Da Tang https://orcid.org/0000-0001-5623-3928

### Data availability

The newly obtained sequences of *Cynanchumpingtaoi* and *C.longhushanense* have been submitted to the NCBI website. The DNA sequence data supporting the findings of this study are available in Suppl. material 2.

## Supplementary materials

Supplementary material 1 List of species names changes within *Cynanchum* in Flora of China Data type xlsx https://binary.pensoft.net/file/1021425This dataset is made available under the Open Database License (http://opendatacommons.org/licenses/odbl/1.0/). The Open Database License (ODbL) is a license agreement intended to allow users to freely share, modify, and use this Dataset while maintaining this same freedom for others, provided that the original source and author(s) are credited.Miao Liao, Si-Jin Zeng, Lin-Ya Zeng, Hai-Jun Yin, Mao-Lin Yan, Cai-Fei Zhang, Guang-Da Tang

Supplementary material 2 List of ingroup and outgroup taxa used in the phylogenetic analyses Data type xlsx Explanation note List of ingroup and outgroup taxa used in the phylogenetic analyses with voucher information (geographic origin, collection, herbarium) and Genbank accession numbers. “*” indicates new sequences; “–” indicates missing sequences; bold GenBank accession numbers indicate connected sequences for *trnL* intron and *trnL-F* intergenic spacer. https://binary.pensoft.net/file/1021426This dataset is made available under the Open Database License (http://opendatacommons.org/licenses/odbl/1.0/). The Open Database License (ODbL) is a license agreement intended to allow users to freely share, modify, and use this Dataset while maintaining this same freedom for others, provided that the original source and author(s) are credited.Miao Liao, Si-Jin Zeng, Lin-Ya Zeng, Hai-Jun Yin, Mao-Lin Yan, Cai-Fei Zhang, Guang-Da Tang

Supplementary material 3 Maximum likelihood tree of *Cynanchum* Data type pdf Explanation note Maximum likelihood tree of *Cynanchum* based on two nuclear regions (nrETS and nrITS) and three plastid markers (*rps16* and *trnL* introns, and *trnL-F* spacer). Bootstrap support values are given for each node. https://binary.pensoft.net/file/1021427This dataset is made available under the Open Database License (http://opendatacommons.org/licenses/odbl/1.0/). The Open Database License (ODbL) is a license agreement intended to allow users to freely share, modify, and use this Dataset while maintaining this same freedom for others, provided that the original source and author(s) are credited.Miao Liao, Si-Jin Zeng, Lin-Ya Zeng, Hai-Jun Yin, Mao-Lin Yan, Cai-Fei Zhang, Guang-Da Tang

Supplementary material 4 Specimens of *Cynanchumhooperianum*, *C.longhushanense*, *C.pingtaoi*, and *C.pulchellum* used for the distribution map Data type docx https://binary.pensoft.net/file/1021428This dataset is made available under the Open Database License (http://opendatacommons.org/licenses/odbl/1.0/). The Open Database License (ODbL) is a license agreement intended to allow users to freely share, modify, and use this Dataset while maintaining this same freedom for others, provided that the original source and author(s) are credited.Miao Liao, Si-Jin Zeng, Lin-Ya Zeng, Hai-Jun Yin, Mao-Lin Yan, Cai-Fei Zhang, Guang-Da Tang

## References

[B1] Ali SI Khatoon S (1982) Genus *Vincetoxicum* von Wolf (Asclepiadaceae) in Pakistan. Pakistan Journal of Botany 14(1): 61–68.

[B2] Backer CA Bakhuizen van den Brink Jr RC (1965) Flora of Java (Spermatophytes only) VOL.II (Angiospermae families 11–160). N.V.P. Noordhoff, 1–713.

[B3] Blume CL (1826) Asclepiadeae. In: Blume CL (Ed.) Bijdragen tot de flora van Nederlandsch Indië, 16de Stuk.Ter Lands Drukkerij, Batavia, 1048–1066. 10.5962/bhl.title.115427

[B4] Chen SF Zhou YQ Chen YR Gu J (2018) fastp: An ultra-fast all-in-one FASTQ preprocessor. Bioinformatics (Oxford, England) 34(17): 884–890. 10.1093/bioinformatics/bty560 30423086 PMC6129281

[B5] Decaisne J (1844) Asclepiadaceae. In: Candolle ALPP (Ed.) Prodromus Systematis Naturalis Regni Vegetabilis vol.8. Victor Masson, Paris, 490–665.

[B6] Delponte GB (1871) Un ricordo botanico del professore Filippo de Filippi ossia cenno intorno alle piante nate dai semi da esso raccolti in Persia e nella China. Memorie della Reale accademia delle scienze di Torino 2(26): 129–168.

[B7] Endress ME Meve U Middleton DJ Liede-Schumann S (2018) Apocynaceae. In: Bittrich V Kadereit JW (Eds) The families and genera of vascular plants 15.Flowering plants. Eudicots. Apiales, Gentianales (except Rubiaceae). Springer, Berlin, 207–411. 10.1007/978-3-319-93605-5_3

[B8] Goyder D (2006) A revision of the genus *Pergularia* L. (Apocynaceae: Asclepiadoideae). Kew Bulletin 61: 245–256. https://www.jstor.org/stable/20443269

[B9] Grubov VI (2000) Conspectus generis *Cynanchum* L. specierum Asiae Centralis. Novosti Sistematiki Vysshikh Rastenii 32: 129–135. [In Russian]

[B10] IUCN (2019) Guidelines for using the IUCN red list categories and criteria. Version 14. Prepared by the standards and petitions subcommittee of the IUCN Species Survival Commission.

[B11] Jin JJ Yu WB Yang JB Song Y de Pamphilis CW Yi TS Li DZ (2020) GetOrganelle: A fast and versatile toolkit for accurate de novo assembly of organelle genomes. Genome Biology 21(1): 241. 10.1186/s13059-020-02154-5 32912315 PMC7488116

[B12] Khanum R Surveswaran S Meve U Liede-Schumann S (2016) *Cynanchum* (Apocynaceae: Asclepiadoideae): a pantropical Asclepiadoid genus revisited. Taxon 65(3): 467–486. 10.12705/653.3

[B13] Kumar S Stecher G Tamura K (2016) MEGA7: Molecular evolutionary genetics analysis version 7.0 for bigger datasets. Molecular Biology and Evolution 33(7): 1870–1874. 10.1093/molbev/msw054 27004904 PMC8210823

[B14] Li PT Gilbert MG Steven WD (1995) Asclepiadaceae. In: Wu ZY Raven PH (Eds) Flora of China, vol 16.Science Press, Beijing and Missouri Botanical Garden Press, St. Louis, Missouri, 189–270.

[B15] Liede S (1996) *Cynanchum*-*Rhodostegiella*-*Vincetoxicum*-*Tylophora* (Asclepiada­ceae): New considerations on an old problem. Taxon 45(2): 193–211. 10.2307/1224660

[B16] Liede S (1997) Subtribes and genera of the tribe Asclepiadeae (Apocynaceae, Asclepiadoideae) - a synopsis. Taxon 46(2): 233–247. 10.2307/1224093

[B17] Liede S (2001) Subtribe Astephaninae (Apocynaceae-Asclepiadoideae) reconsidered: New evidence based on cpDNA spacers. Annals of the Missouri Botanical Garden 88(4): 657–668. 10.2307/3298638

[B18] Liede S Kunze H (1993) A descriptive system for corona analysis in Asclepiadaceae and Periplocaceae. Plant Systematics and Evolution 185(3–4): 275–284. 10.1007/BF00937663

[B19] Liede S Kunze H (2002) *Cynanchum* and the Cynanchinae (Apocynaceae – Asclepiadoideae): A molecular, anatomical and latex triterpenoid study. Organisms, Diversity & Evolution 2(3): 239–269. 10.1078/1439-6092-00045

[B20] Liede S Täuber A (2002) Circumscription of the genus *Cynanchum* (Apocynaceae-Asclepiadoideae). Systematic Botany 27(4): 789–800.

[B21] Liou YX Yang HL Yao YY (1992) Flora In Desertis Reipublicae Populorum Sinarum, Tomus 3. Science Press, Beijing, 508 pp. [In Chinese]

[B22] Liu B (2023) *Vincetoxicum* in Catalogue of Life China: 2023 Annual Checklist, Beijing, China. http://www.sp2000.org.cn/browse/browse_this_taxa/b6d13149-b378-4b3a-9f4d-7942e74b600f

[B23] Markgraf F (1972) Asclepiadaceae. In: Tutin TG Heywood VH Burges NA Moore DM Valentine DH Walters SM Webb DA (Eds) Flora Europaea, Volume 3.Diapensiacea to Myoporaceae. Cambridge University Press, Cambridge, 70–73.

[B24] Miller MA Pfeiffer W Schwartz T (2010) Creating the CIPRES science gateway for inference of large phylogenetic trees. Proceedings of the gateway computing environments workshop (GCE) New Orleans, LA, USA, 1–8. 10.1109/GCE.2010.5676129

[B25] POWO (2024) . Plants of the World Online. Facilitated by the Royal Botanic Gardens, Kew. Published on the Internet. http://www.plantsoftheworldonline.org/ [Retrieved 24 February 2024]

[B26] Qiu SX Li DZ Zhang ZX Zhou J Wu ZY (1989) Chemotaxonomy of *Cynanchum* and its allied genera with notes on the generic characteristics of *Vincetoxicum*. Yunnan Zhi Wu Yan Jiu 11(1): 41–50. [In Chinese]

[B27] Rambaut A (2012) . FigTree version 1.4.0. http://tree.bio.ed.ac.uk/software/figtree/

[B28] Rapini A van den Berg C Liede-Schumann S (2007) Diversification of Asclepiadoideae (Apocynaceae) in the New World. Annals of the Missouri Botanical Garden 94(2): 407–422. 10.3417/0026-6493(2007)94[407:DOAAIT]2.0.CO;2

[B29] Shen JY Ma DX Wang WG Shi JP (2019) *Cynanchumbrevipedunculatum*, a new species of Apocynaceae from Yunnan, China. Taiwania 64(3): 217–220. 10.6165/tai.2019.64.217

[B30] Stamatakis A (2014) RAxML version 8: A tool for phylogenetic analysis and post-analysis of large phylogenies. Bioinformatics (Oxford, England) 30(9): 1312–1313. 10.1093/bioinformatics/btu033 24451623 PMC3998144

[B31] Thiers B (2022) Index Herbariorum: A global directory of public herbaria and associated staff. New York Botanical Garden’s Virtual Herbarium. http://sweetgum.nybg.org/science/ih/ [Accessed 2.11.2022]

[B32] Tsiang Y Li PT (1977) Florae Reipublicae Popularis Sinicae, Tomus 63. Science Press, Beijing, 617 pp. http://www.iplant.cn/frps/vol/63 [In Chinese]

[B33] Wallich N (1831) Plantae Asiaticae rariores; or, Descriptions and figures of a select number of unpublished east Indian plants VOL. II. Treuttel and Würtz, London, 1–86. 10.5962/bhl.title.468

[B34] Wan Y (1983) A new species of *Raphistemma* (Asclepiadaceae) from Guangxi. Guangxi Zhi Wu 3(3): 197–199. [In Chinese]

[B35] Woodson Jr RE (1941) The North American Asclepiadaceae. I. Perspective of the Genera. Annals of the Missouri Botanical Garden 28(2): 193–244. 10.2307/2394270

[B36] Xu WB Xia BS Wu JQ Chen YX Shen JY (2021) *Cynanchumhubeiense* (Apocynaceae), a new species from Hubei, China. Taiwania 66(1): 53–56. 10.6165/tai.2021.66.53

[B37] Yamazaki T (1968) Supplement of the flora of Ryukyu and Formosa (2). Shokubutsu Kenkyu Zasshi 43(6): 168–170. [In Japanese]

