# Supplementary figures and images for: ﻿A new species and a replacement name in Cynanchum (Apocynaceae, Asclepiadeae) from China
Source: PhytoKeys. 2024 Apr 8;241:49–63. doi: 10.3897/phytokeys.241.111499 (PMC11019257; doi:10.3897/phytokeys.241.111499)

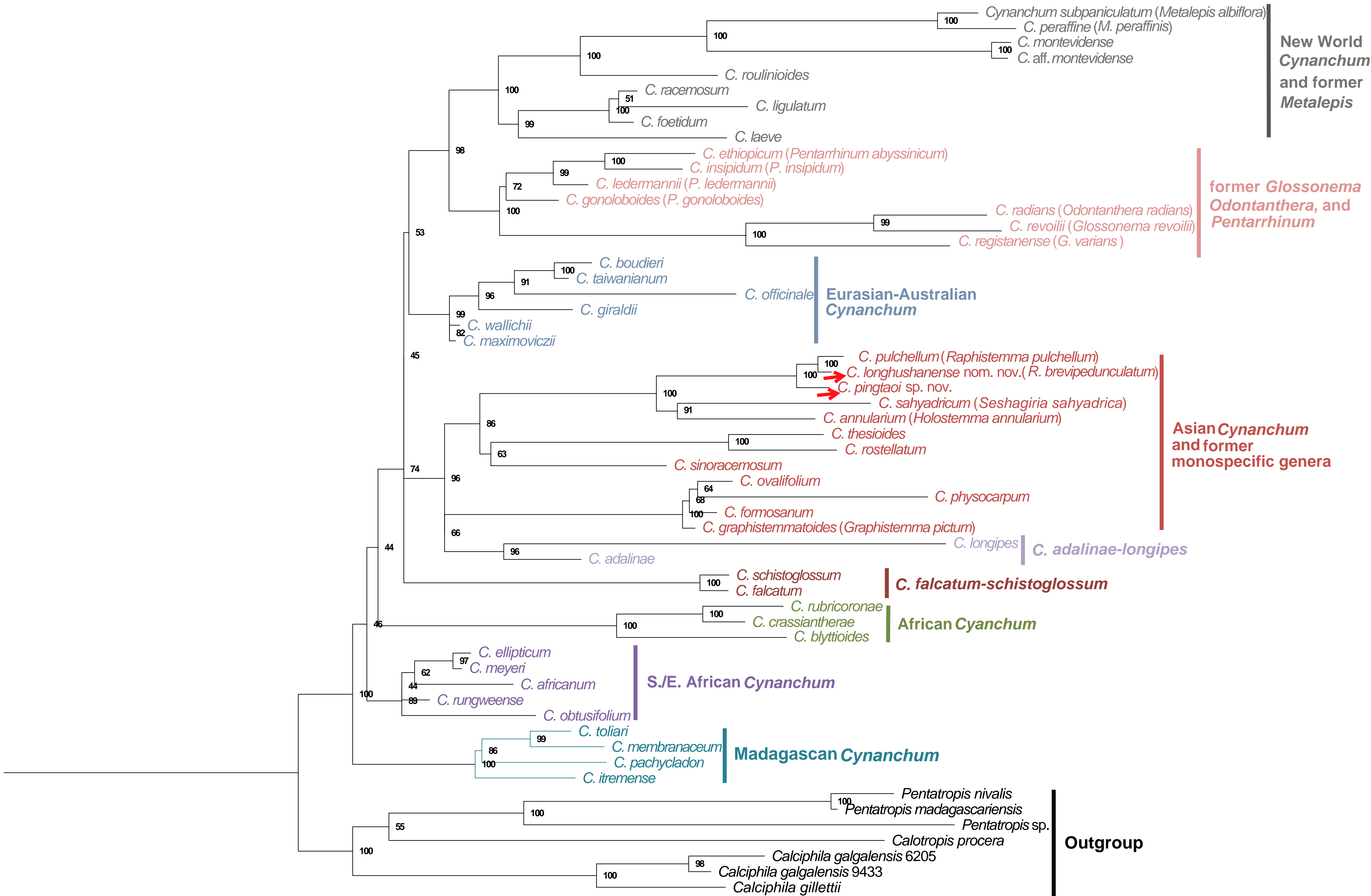

Supplement: Supplementary material 3 — Maximum likelihood tree of Cynanchum [file phytokeys-241-049_article-111499__-s003.pdf]
