# Supplementary material for: ﻿A new species and a replacement name in Cynanchum (Apocynaceae, Asclepiadeae) from China
Source: PhytoKeys. 2024 Apr 8;241:49–63. doi: 10.3897/phytokeys.241.111499 (PMC11019257; doi:10.3897/phytokeys.241.111499)
Supplement: Supplementary material 4 — Specimens of Cynanchumhooperianum, C.longhushanense, C.pingtaoi, and C.pulchellum used for the distribution map [file phytokeys-241-049_article-111499__-s004.docx]

**Specimens of *Cynanchum hooperianum*, *C. longhushanense*, *C. pingtaoi*, and *C. pulchellum* used for the distribution map**

***C. hooperianum* CAMBODIA.** Battambang, 23 Oct. 1928, *unknown collector s.n.* (P[P04918797]); Krong Kampot, 18 Oct. 1903, *M. Geoffray 140* (P[P04918794]); **INDONESIA.** Java, without collection date, *unknown collector s.n.* (K[K000894599]); Java, without collection date, *H. Thomas. 14* (K[K000894601]); Java, without collection date, *M. Blume s.n.* (P[P04918780]); Java, without collection date, *C.L. von Blume s.n.* (U[U.1096463]); **LAOS.** Champasak Province, Khong District, Sahong Island, central part, east of the village, Mekong River, 60 m a.s.l., 15 Oct. 1997, *J.F. Maxwell 97-1181* (L[L.3738856]); **THAILAND.** Kanchanaburi Province, Sai Yok District, Mahidol University, Kanchanaburi Campus, east side, Doi Hin area, c. 5 km north of Soi Yok, Loom Soom Subdistrict, 175 m a.s.l., 18 Dec. 2005, *J.F. Maxwell 05-719* (L[L.3738857]); Kanchanaburi Province, Sai Yok District, Mahidol University, Kanchanaburi Campus, summit of a rugged limestone hill at Dtairattanarahm Temple, c. 5 km north of Sai Yok, Loom Soom Subdistrict, 225 m a.s.l., 11 Sept. 2005, *J.F. Maxwell 05-475* (L[L.3738859]); Nakorn Sawan Province, Takli District, near cave entrance 400 m north of headquarters along a nature trail, Doi Kun Ming/Chon Duea, Tahm Pet Tahm Tong Forest Park., 80 m a.s.l., 18 Oct. 2006, *M. van de Bult 902* (L[L.3738858]).

***C. longhushanense* CHINA. Guangxi:** Baise City, Long'lin County, near Tiansheng Bridge 8 Nov. 1983, *Zi En Chao 1824* (HIB[HIB0101976]); Nanning City, Long'an County, Longhushan Nature Reserve, 27 Oct. 1981, *X.H. Lu & C.C. Huang 11776* (GXMG[GXMG0071479]); Nanning City, Long'an County, Pingshan Village, 10 Oct. 1977, *Longan Investigation Team 2-040* (GXMI[GXMI031735]); Nanning City, Long'an County, Longhushan Nature Reserve, 14 Nov. 1982, *Y. Wan & Rui-Ju Liu 82430* (paratype: GXSP [GXSP0000039]); Nanning City, Long’an County, Longhushan Nature Reserve, open woods, 2 Jul. 1981, *D.H. Tan 82329* (GXSP[GXSP0000038]; Nanning City, Long'an County, Longhushan Nature Reserve, 25 Jun. 2021, *Miao Liao LM78* (IBSC).

***C. pingtaoi* CHINA. Yunnan:** Dehong Dai and Jingpo Autonomous Prefecture, Ruili City, Nongdao Town, Tongbiguan Provincial Nature Reserve, 24 Aug. 2020, *Si-Jin Zeng & Lin-Ya Zeng SJ4825* (IBSC[IBSC1009908]).

***C. pulchellum* BANGLADESH.** Sylhet, without collection date, *De Silva, F. Cat. no. 1310* (K[K001112693]); Sylhet, without collection date, *N. Wallich 1310c* (M[M-0175099]); **CAMBODIA.** forest near Angkor, Jun. 1909, *A.C. d' Alleizette 4766* (L[L.2716656]); **CHINA. Yunnan:** Dehong Dai and Jingpo Autonomous Prefecture, Mang City, Zhefang Town, Manghai Village 24°6′N, 98°12′E, 17 Aug. 1976, *S.J. Pei 14001* (HITBC[HITBC098324], HITBC[HITBC035610]); Honghe Hani and Yi Autonomous Prefecture, Jinping Miao, Yao and Dai Autonomous County, 400 m a.s.l., 20 Aug. 1951, *P.I. Mao 619* (KUN[KUN309334]); Lincang City, Zhenkang County, Nanting Riverside, 800 m a.s.l., 17 Oct. 1958, *T.P. Zhu 0151* (KUN[KUN309340]); Honghe Hani and Yi Autonomous Prefecture, road from Mengzi City to Yuanyang County, Fengchunling Township, 6 Apr. 1941, *Liou 018538* (PE[PE01025879]); Pu'er City, Jinggu Dai and Yi Autonomous County, Zhengxing Town, Boyun Village, 23 Sept. 1985, *S.C. Ho 85474* (IBSC[IBSC0521197]); Tengchong City, Puchuan Township, Xiajia Village, 1300~1650 m a.s.l., 15 Oct. 1960, *W.Q. Yin 60-1463* (KUN[KUN309333]); Xishuangbanna Dai Autonomous Prefecture, Menghai County, Gelanghe Township, Heilongtan Village, 1900 m a.s.l., Jul. 1936, *C.W. Wang 76267* (IBSC[IBSC0521195], PE[PE01025874], KUN[KUN309336], PE[PE01025875], WUK[WUK0047379]); Xishuangbanna Dai Autonomous Prefecture, Jinghong City, Damengyang, 950 m a.s.l., 18 Aug. 1936, *C.W. Wang 77690* (IBSC[IBSC0521196], KUN[KUN309337], NAS[NAS00209757], PE[PE01025873], PE[PE01025876], WUK[WUK0045748]); Xishuangbanna Dai Autonomous Prefecture, Jinghong City, Xiaomengyang, 1100 m a.s.l., Aug. 1936, *C.W. Wang 75774* (IBSC[IBSC0521194], NAS[NAS00209756], PE[PE01025870], PE[PE01025877], WUK[WUK0047038]); Xishuangbanna Dai Autonomous Prefecture, Jinghong City, Xiaomengyang, 1100 m a.s.l., Aug. 1936, *C.W. Wang 75775* (KUN[KUN309338]); Xishuangbanna Dai Autonomous Prefecture, Jinghong City, 22°18′N, 100°54′E, 18 Aug. 1977, *G.D. Tao et al. 17733* (HITBC[HITBC098319]); Xishuangbanna Dai Autonomous Prefecture, Menghai County, Mengzhe town, 1280 m a.s.l., Jun. 1936, *C.W. Wang 75382* (PE[PE01025871], PE[PE01025872]); Xishuangbanna Dai Autonomous Prefecture, Mengla County, Xiangming Township, Manzhuang Village, 900 m a.s.l., 1 Dec. 1990, *Y.H. Li et al. 1018* (KUN[KUN1267340]); **CHINA. Guangxi:** Baise City, Tianlin County, 600 m a.s.l., 26 Jun. 1981, *D.H. Qin 26441* (GXMI [GXMI031736]); Baise City, Xilin County, 25 Jun. 1936, *H.H. Su 67873* (PE[PE01025878]); **INDIA.** Aassam, Goalpara, without collection date, *unknown collector s.n. Cat. no. 1310* (K[K001112692]); Aassam, Goalpara, without collection date, *Buch.-Ham 749* (E[E00649591]); Aassam, without collection date, *C. Jenkins s.n.* (P[P04918806], P[P04918808]); Aassam, without collection date, *C. Jenkins 186* (P[P04918812]); Hab. Sikkim. Regio trop, without collection date, *J.D. Hooker s.n.* (L[L.2716658]); West Bengal, Howrah district, Howrah city, Shibpur, Acharya Jagadish Chandra Bose Indian Botanic Garden, without collection date, *unknown collector s.n. Cat. no. 1310* (K[K000639615]); **LAOS.** Champasak Province, Khong District, Xang Island, east side, near the village, 60 m a.s.l., 10 Oct. 1997, *J.F. Maxwell 97-1112* (L[L.3738863]); **MALAYSIA.** Perak, Jul. 1886, *Dr King s.n.* (E[E00649593]); **MYANMAR.** Tanintharyi Region, Dawei, without collection date, *W. Gomez. Cat. no. 1310* (K[K001112695]); Thazi-Kalaw, Kalaw, 1219 m a.s.l., Sept. 1917, *C.G. Rogers 1041* (E[E00269848]); Yangon, 1826, *N. Wallich Cat. no. 1310* (K[K001112694]); **NEPAL.** Gandaki Province, pokhara, 1067 m a.s.l., 15 Aug. 1954, *J.D.A. Stainton*, *W. Sykes & J. Williams 6897* (E[E00649587]); Sudurpashchim Province, Kosi Zone, Bajhang District, Chainpur, 762 m a.s.l., 5 Sept. 1956, *J.D.A. Stainton 1547* (E[E00649592]); central Nepal, northeast of Kathmandu, Gokarna Forest, 22 Sept. 1963, *H. Hara, H. Kanai, S. Kurosawa s.n.* (TI[TI6302630]). **THAILAND.** Chiang Mai Province, Chiang Dao District, along the Mae Dtang River, near Muang Kawng town, 650 m a.s.l., 1 Apr. 1995, *J.F. Maxwell 95-312* (L[L.3738864]); Kanchanaburi Province, Road between Kritee and Meung Chah, 15°1'59"N, 98°45'0"E, 700 m a.s.l., 7 Oct. 1973, *R. Geesink & C. Phengklai 6222* (L[L.2716651]); Lampang Province, Mae Ta District, Mawn Huay Rai (hill), Mae Tahn Village, Sahn Dawk Gayo Subdistrict, 400 m a.s.l., 18 Oct. 2010, *J.F. Maxwell 10-31* (L[L.2058963]); Lampang Province, Muang Bahn (Pan) District, Jae Sawn National Park, east side, along the dirt road to Mae Jam Village, below Huay Miang (Mong) Village., 650 m a.s.l., 23 Sept. 1996, *J.F. Maxwell 96-1259* (L[L.3738862]); Lampoon Province, Mae Tah District, Doi Kuhn National Park, below Yaw, near canteen, along the main road., 825 m a.s.l., 29 Sept. 2001, *P. Palee 498* (L[L.3738865]); Muang District, Khao Yai National Park, 750 m a.s.l., Jul.-Aug. 1966, *Kai Larsen, T. Smitinand & E.Warncke 392* (P[P04918809]); Phang Nga Province, Ko Kho Khao, 7 Dec. 1965, *B. Sangkhachand 1194* (L[L.2716652]); Sakon Nakhon, 27 Jun. 1932, *M.C. Lakshnakara 1008* (L[L.2716655]); Phitsanulok, Chat Trakan, Phu Miang-Phu Thong Wildlife Sanctuary, 17°30' 34'' N, 100°48' 50'' E, 345 m a.s.l., 12 Oct. 2009, *D.J. Middleton, P. Karaket, S.Lindsay, T. Phutthai, S. Suddee & N. Tetsana 5103* (E[E00392987]); Satun, Khuan Don, beside road just outside Thale Ban National Park, 6°42' 48'' N, 100°10' 1'' E, 105 m a.s.l., 9 Sept. 2010, *D.J. Middleton, K. Bunpha, P. Karaket, S. Lindsay, T. Phutthai & S. Suddee 5359* (E[E00428235]); Tak, Phop Phra, Km 50 Mae Sot-Umphang Road., 25 Aug. 2008, *P. Karaket 4* (L[L.3738860], L[L.3738861]); **VIETNAM.** Thanh Hoa province, Thach Thand District, Cuc Phuong Np, Thanh Yenh Commune, 20°14.63' N, 105°40.52' 50'' E, 150 m a.s.l., 27 Feb. 2001, *N.Manh. Cuong* & *M.V. Xinh NMC_1323* (P[P04918819]).
